# Extended Focused Assessment with Sonography Accuracy at a Community Level III Trauma Center

**DOI:** 10.51894/001c.164406

**Published:** 2026-08-01

**Authors:** Caitlyn Cross, Hussein Chams, Siddhi Upadhyaya, Jason Mikhail, Dennis Smythe, Richard Spinale

**Affiliations:** 1 Garden City Hospital

## 50

### Introduction

At our community hospital Level III trauma center, Extended Focused Assessment with Sonography for Trauma (eFAST) examinations are the most utilized imaging method for trauma activations. Due to this imaging technique being used more prolifically than computed tomography (CT), the eFAST exams were analyzed to evaluate their accuracy and correlation with CT findings.

### Objective

The goal of this analysis was to improve the interpretation and accuracy of eFast as this imaging technique is the main imaging method obtained at this facility.

### Methods

A total of 45 eFAST examinations were reviewed and assessed for the quality of ultrasound technique, accuracy of interpretation, and correlation with CT scan findings when CT imaging was obtained.

### Results

Of the 45 ultrasounds reviewed, only 25 (55%) of examinations were determined to be accurately obtained and correctly interpreted. Six examinations were both technically adequate and correctly interpreted, with findings that corresponded to negative CT scans. One examination demonstrated a positive finding in the splenorenal view on eFAST; however, subsequent CT imaging revealed that the finding corresponded to large renal cysts rather than free fluid. In two cases, the eFAST examinations were initially interpreted as negative, but upon reevaluation the images were found to be inadequate due to incomplete visualization of required views. Additionally, several pericardial effusions were identified on eFAST examination, while multiple CT scans demonstrated mild ascites that were not visualized on eFAST. These results indicate that the accuracy of the exams is dependent on the experience of the user. Additionally, the most accurate exams were performed by attending physicians, whereas the least accurate eFAST exams were performed by residents in training.

### Discussion

These findings highlight the importance of proper eFAST examination technique and adequate image acquisition to ensure diagnostic accuracy in trauma evaluation. With these findings, providers must now follow an eFAST check off list that ensures the examiner accurately obtains the required views for accurate interpretation.

